# L-Tyrosine Limits Mycobacterial Survival in Tuberculous Granuloma

**DOI:** 10.3390/pathogens12050654

**Published:** 2023-04-28

**Authors:** Yaxian Gao, Jiaqing Li, Xinya Guo, Liru Guan, Jie Wang, Xiaochen Huang, Wenjuan Wang, Hua Yang

**Affiliations:** 1 The Key Laboratory of Environmental Pollution Monitoring and Disease Control, Ministry of Education, School of Public Health, Guizhou Medical University, Guiyang 550000, China; 2 Shanghai Key Laboratory of Tuberculosis, Shanghai Pulmonary Hospital, Tongji University School of Medicine, Shanghai 200433, China

**Keywords:** mycobacterium, tuberculous granuloma, amino acid, L-tyrosine, zebrafish, treatment

## Abstract

Caused by the intracellular pathogen *Mycobacterium tuberculosis* (Mtb), tuberculosis (TB) remains a massive global public health issue. A well-known and key TB trait is caseous necrotic granuloma, which allows mycobacteria to reactivate and disseminate, thus confounding TB eradication programs. Amino acid (AA) metabolism is key to regulating immune responses in Mtb infections; however, it is currently unclear if AAs can be used to treat tuberculous granulomas. Here, we screened 20 proteinogenic AAs using a *Mycobacterium marinum*-infected zebrafish granuloma model. Only L-tyrosine simultaneously reduced *Mycobacterium marinum* (*M*. *marinum*) levels in zebrafish larvae and adults and inhibited intracellular pathogen survival levels. Mechanistically, L-tyrosine significantly upregulated interferon-γ (IFN-γ) expression in *M*. *marinum* -infected zebrafish adults but not in larvae. Using N-acetylcysteine (NAC) to inhibit reactive oxygen species (ROS), L-tyrosine appeared to inhibit Mtb intracellular survival by promoting ROS production. Thus, L-tyrosine as a non-essential AA may reduce mycobacterial survival in both macrophages and tuberculous granulomas. Our research provides a platform for the clinical development of AAs for active or latent TB patients infected with drug-sensitive or drug-resistant Mtb.

## 1. Introduction

Caused by the intracellular pathogen *Mycobacterium tuberculosis* (Mtb), tuberculosis (TB) remains the world’s deadliest infectious disease. In 2021, an estimated 1.6 million global deaths were caused by TB, up from approximately 1.5 million in 2020 and 1.4 million in 2019, and returning to 2017 levels [1]. In 2020, TB care and services were severely disrupted and progress against the disease set back by the COVID-19 pandemic [2]. Although first-line drugs or the newly developed bedaquiline can efficiently treat active TB, drug resistance often rapidly develops and attenuates clinical efficacy [3,4]. One well-known TB clinical trait is caseous necrotic granuloma, which includes organized macrophages and other immune cell aggregates and provides niches for Mtb to derive nutrients or evade anti-TB therapies, thereby providing mycobacteria sources for subsequent re-emergence [5,6,7]. Additionally, tuberculous granuloma structures are largely immune to drug penetration and bacterial elimination, thus generating clinical/therapeutic issues and confounding TB eradication programs [8]. Consequently, new strategies, such as novel host-directed therapies (HDT), are required to eradicate Mtb in granulomas and prevent mycobacterial spread.

Amino acid (AA) metabolism helps regulate immune responses to Mtb infections [9,10]. Some AAs or downstream metabolites appear to function as anti-microbial agents [11,12]. In recent years, both L-arginine (L-Arg) and L-tryptophan (L-Trp) have been proposed as TB therapies [9,13]. L-Arg is used by macrophages to generate nitrogen monoxide (NO) via inducible nitric oxide synthase [14]. While L-Arg is required for immune cell action, in vitro and in vivo supplementation have been proposed as potential therapeutic or vaccine adjuvants, but it remains unclear if L-Arg can limit Mtb survival in granulomas [15,16,17,18]. For its survival, Mtb requires the tryptophan biosynthetic pathway [19], but interferon-γ (IFN-γ) induces an indoleamine 2, 3-dioxygenase (host enzyme) isoform, which converts tryptophan to N-formylkynurenine, thus depleting tryptophan levels and its antimicrobial effects [9,20]. Therefore, the therapeutic potential of L-Trp supplementation, with respect to Mtb infection, remains unexplored [9], and it is unclear whether AA supplementation can control tuberculous granulomas.

Zebrafish infected by *M*. *marinum* are ideal subjects to study host immune responses to mycobacterial infections. *M*. *marinum* is closely related to Mtb; their genomes contain approximately 3000 orthologous proteins of which 85% are identical at the AA level [21,22]. Upon systemic zebrafish infection with *M*. *marinum*, caseous granulomas form and are histologically similar to tuberculous granulomas in humans after Mtb infection [23]. Furthermore, zebrafish larvae can be used to highlight initial granuloma development in terms of innate immunity [24]. Zebrafish can also be used for large-scale screening studies due to their small size and high offspring levels. Fish are transparent at larval stages, allowing for the real-time tracking of host-pathogen interactions [25]. Similarly, the maturation of the adaptive immune system is not complete until approximately 4–6 weeks post-fertilization; therefore, innate immunity mechanisms can be studied prior to this stage [26]. In our study, we exploited these advantages to screen for AAs that controlled bacterial burden and the proportion of high-burden or necrotic granulomas. We observed that L-Tyr supplementation exerted therapeutic roles by limiting mycobacterial survival in tuberculous granulomas; thus, our work contributes valuable insights to conventional therapies.

## 2. Materials and Methods

### 2.1. Bacterial Cells/Strains

We constructed an H37Rv reporter strain expressing a live–dead plasmid (a gift from Dr. Bryan Bryson (Department of Biological Engineering, MIT, MA, USA). The strain expresses a long-lived red fluorescent protein, which marked all bacteria. Bacteria responding to tetracycline and expressing green fluorescent protein (GFP) were considered transcriptionally active and used as viability proxies (live bacteria) [27]. The reporter strain was cultured in Middlebrook 7H9 medium (Difco/Becton Dickson, Franklin Lakes, NJ, USA) with 10% ADC [5% bovine serum albumin, 2% dextrose, and 5% catalase] and 0.05% Tween-80 (Sigma-Aldrich, St. Louis, MO, USA). Then, 50 µg/mL hygromycin B. tdTomato-labeled *M*. *marinum* (a gift from Dr. Stefan Oehlers, Infectious Diseases and Immunology, University of Sydney) was cultured to mid-log phase (Optical Density at 600 nm = 0.6) in 7H9 plus hygromycin B (50 µg/mL). For preservation, aliquots were mixed with 20% glycerol and 7H9 medium and stored at −80 °C. Stock CFUs/mL were determined by plating serial dilutions on 7H10 agar plates plus 10% ADC and used for zebrafish infections.

Mouse peritoneal macrophages (MPMs) were isolated from C57BL/6 mice and cultured as previously described [28]. Mice were intraperitoneally injected with 2 mL of Brewer’s thioglycollate medium (4%) (Sigma-Aldrich). After 72 h, mice were humanely sacrificed via cervical dislocation. Peritoneal cavity cells were flushed out into tubes for cell counting/plating using 10 mL Roswell Park Memorial Institute (RPMI) 1640 medium/mouse (Thermo, Waltham, MA, USA). Adherent MPMs for analyses were grown in RPMI-1640 medium plus 10% (*v*/*v*) heat-inactivated fetal bovine serum (Thermo) and 100 U/mL pen/strep (Thermo). 

### 2.2. Zebrafish Strains and Maintenance

The Animal Experiment Administration Committee of Shanghai Pulmonary Hospital (K18-033) approved all animal protocols. Investigations were conducted according to the China National Research Council’s Guide for Care and Use of Laboratory Animals. Zebrafish handling complied with animal welfare regulations and fish were maintained according to standard protocols available at zfin.org, (accessed on 1 December 2021). 

Wild-type (WT) adult zebrafish were purchased from EzeRinka (Nanjing, China), reared in a recirculating system (Qingdao Elvin Marine Technology Co., Ltd., Qingdao, China), and transferred to a flow-through system for infection studies. Approximately 10 fish were housed in 3 L tanks (conditions: pH ~ 7.4; water temperature ~ 28 °C; conductivity ~ 1500 μS). Fish were maintained in a 14 h/10 h light/dark cycle and fed twice a day. 

Larvae: Family cross embryos were collected and grown (28 °C) in the dark in egg water (60 µg/m Instant Ocean Sea Salts; Sera Marin). To prevent pigment formation, N-phenylthiourea (0.0045%) (Sigma-Aldrich) was added.

### 2.3. AAs and Reagents

L-arginine (L-Arg), L-aspartic acid (L-Asp), L-glycine (L-Gly), L-glutamine (L-Gln), L-glutamic acid (L-Glu), L-isoleucine (L-Ile), L-lysine (L-Lys), L-proline (L-Pro), L-serine (L-Ser), L-tryptophan (L-Trp), L-threonine (L-Thr), L-valine (L-Val), L-histidine (L-His), L-alanine (L-Ala), L-asparagine (L-Asn), L-cysteine (L-Cys), L-leucine (L-Leu), L-methionine (L-Met), L-phenylalanine (L-Phe), and L-tyrosine (L-Tyr) disodium salt hydrate were provided by Sigma. As L-Tyr was practically insoluble in water, we used L-Tyr disodium salt hydrate and calculated its effective concentration for treatments based on molecular weight. The reactive oxygen species (ROS) inhibitor N-acetylcysteine (NAC) and rifampicin (RFP, first-line anti-TB drug) were purchased from MCE (MedChemExpress, Monmouth Junction, NJ, USA). 

### 2.4. Larvae Infections, Treatments, and Image Analyses

At 2 days post-fertilization (dpf), larvae were anesthetized with tricaine, injected into the duct of Cuvier with approximately 200 CFU tdTomato-labeled *M*. *marinum*, and transferred to 48-well plates with 200 µL of egg water/well. At 1 day post-infection (dpi), larvae were treated with AAs or RFP until 4 dpi. Larvae were then exposed to AAs for 40 h, with compounds added daily. After 40 h, three larvae were extracted in 1 mL of Trizol to determine inflammatory factor expression.

After 40 h exposure, fluorescence microscopy (Leica DMi8, Weztlar, Germany) was used to image and assess AA treatment efficacy in limiting mycobacterial survival in larval tail regions. We quantified bacterial burden (Image J software, Madison, WI, USA) by measuring integrated fluorescent intensity levels of labeled bacteria. 

### 2.5. Acute Toxicity Assays

We treated WT embryos at 2 dpf with different AA concentrations. Feeding commenced at 5 dpf with a dry larval diet (Larva Z Plus, EzeRinka, Nanjing, China) twice a day, and embryo death or development was monitored on a daily basis until 9 dpf.

### 2.6. Adult Zebrafish Infections and Treatments

Fish were anesthetized using ethyl 3-aminocrotonate methanesulfonate (0.016%) (Sigma-Aldrich) and infected via the intraperitoneal injection of approximately 400 CFUs of bacterial strains. Adult zebrafish were orally administered each AA (20 in total) at effective concentrations every day (Table S1) for 1 week before infection and for 2 weeks thereafter. As a positive control, at 7 dpi, other fish were orally administered with RFP (10 mM) every day until 14 dpi. To assess bacterial burden in whole fish post-infection, fish (at 14 days) were terminally anesthetized using tricaine (0.4%) and homogenized in 1.0 mL of phosphate-buffered saline (PBS), and dilutions were plated onto solid 7H10 agar. We plated three or five condition, and bacterial counts were enumerated. Tails from five fish were RNA extracted and quantitated by real-time polymerase chain reaction (RT-PCR) to determine inflammatory factor expression. For histology, fish were fixed in neutral-buffered paraformaldehyde solution (4%) for 72 h, and paraffin was embedded, sectioned, and stained with hematoxylin and eosin (H&E) or Ziehl–Neelsen stains. Acid-fast granuloma staining identified acid-fast *bacilli* and bacterial burden, while H&E staining indicated necrotic granuloma regions. Tuberculous granulomas from different *M. marinum*-infected adult zebrafish were scored for *M. marinum* burden; < or >ten bacteria, and necrotic granuloma percentages were quantified using staining data. Macrophage aggregates containing nuclear and cytoplasmic debris represented necrotic granulomas, while non-necrotic granulomas were composed of epithelioid macrophages with clear areas between cells [29]. Total granulomas/strain were counted and were represented as “n”, while granulomas between different groups represented *M*. *marinum*-infected zebrafish pathology [28].

### 2.7. Cell Infections and Image Analyses

MPMs (2.5 × 10^5^ cells/well) were seeded in 48-well culture plates containing RPMI-1640 medium. Cells were pretreated overnight with AAs (10 μM) following infection with 100 ng/mL anhydrotetracycline-induced live–dead H37Rv reporter strain for 3 h or 24 h (multiplicity of infection = 5). Cells washed in PBS were then fixed in paraformaldehyde (4%), and imaging was performed as follows: MPMs and resident Mtb cells were imaged under fluorescence microscopy (Leica DMi8). In bright-field, individual cells were demarked. The cell background per macrophage was measured (Image J software) at 580 nm (mCherry) and 525 nm (GFP). Pixel intensities at four standard deviations (SDs) above average cell background signals (580 nm channel) were deemed positive for Mtb mCherry constitutive signals, while pixel intensity values at two SD units above average cell backgrounds (525 nm channel) were deemed positive for Mtb GFP live signals. Pixels positive for only Mtb mCherry signals were GFP measured. Bacterial survival rates were determined by dividing total intracellular GFP-positive signals by total intracellular mCherry-positive signals [27]. 

### 2.8. Quantitative RT-PCR (qRT-PCR)

From adult zebrafish tails or whole larvae, we extracted RNA using Trizol (TaKaRa), and 1 μg total RNA was used for cDNA synthesis (PrimeScript reverse transcription kit, ABclonal, Wuhan, China), after which qRT-PCR was performed using a SYBR Green kit (ABclonal). We used the 2^−ΔΔCt^ approach to analyze relative gene expression, which was normalized to β-actin for all the samples and then normalized to infected and untreated fish to arrive at a value of relative expression. Gene-specific primers are shown (Table 1).

### 2.9. Statistical Analyses

All data were processed in GraphPad Prism 8.0. Results were represented as the mean ± standard error of the mean. Statistically significant differences between two groups were identified using two-tailed unpaired Student’s *t*-tests. Populations were compared using Fisher’s exact tests. *p* < 0.05 indicated statistical significance.

## 3. Results

### 3.1. L-Asp, L-Thr, and L-Tyr Inhibit M. marinum Proliferation in Larva Granulomas

**Figure 1:**
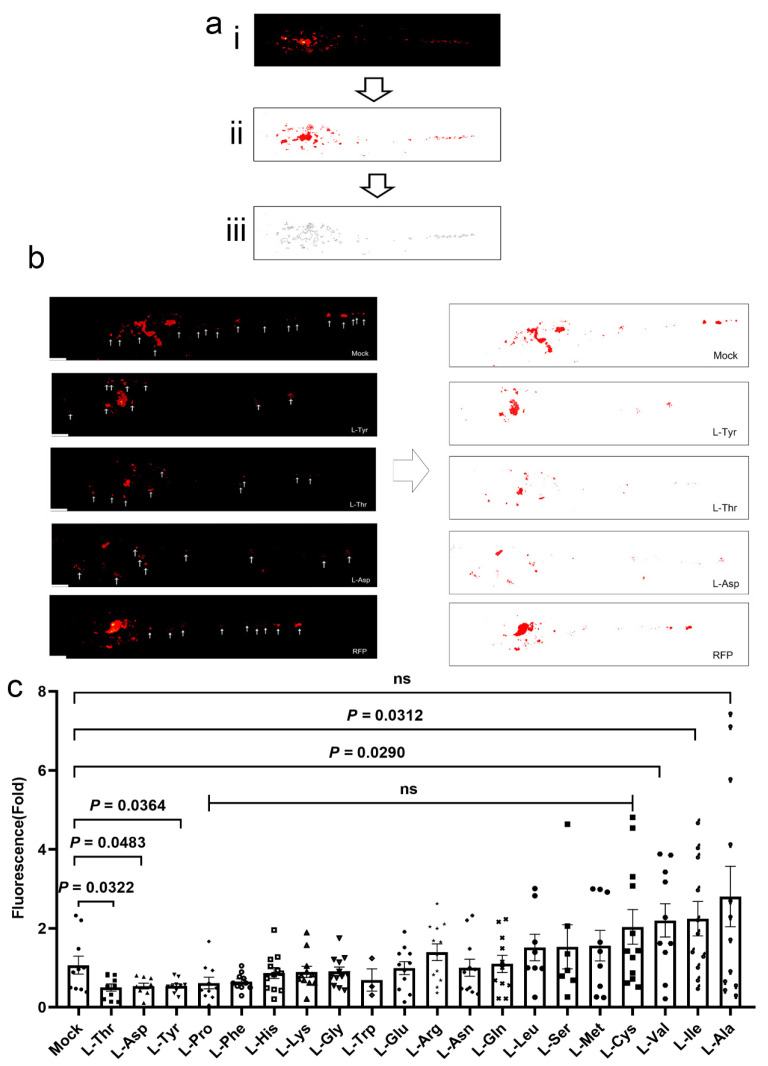
L-Asp, L-Thr, and L-Tyr inhibit *M*. *marinum* proliferation in zebrafish larvae. (**a**) Process used to calculate bacterial burden in larvae. Original image (i); image after fluorescence pixelation—red pixels represent relative bacterial burden size (ii); area calculation (iii). (**b**) Images showing *M*. *marinum* burden distribution in wild-type zebrafish larva treated with control, RFP, L-Asp, L-Tyr, and L-Thr (10 μM) for 3 days. White arrows show *M*. *marinum* infection areas. (**c**) Bacterial burden in *M*. *marinum*-infected larvae treated with control, RFP (10 μM), or amino acids (10 μM) for 3 days. Individual values are indicated in a scatter dot plot in (**c**). Student’s *t*-tests (**c**) were used. *p* < 0.05 was statistically significant. Scale bar = 200 μm in (**b**).

To examine if AA supplementation controlled bacterial burden in tuberculous granulomas, we investigated embryonic zebrafish. We evaluated the antimicrobial effects of 20 AAs (uniform concentrations) on *M*. *marinum*-infected larvae. As shown (Figure 1a), larvae were successfully infected with fluorescent *M*. *marinum*. Larvae were then cultured with mock, RFP (10 μM) [30] (positive control) and AAs (10 μM) for 3 days, and relative fluorescence intensities were monitored to quantify bacterial burden (Figure 1a). When compared to the sibling controls and RFP, only L-Asp, L-Thr, and L-Tyr supplementation significantly inhibited *M*. *marinum* bacterial burden in larvae (Figure 1b,c). Importantly, 15 AAs and RFP exerted no significant effects on *M*. *marinum* proliferation, including L-Arg and L-Trp, while 2 AAs (L-Val and L-Ile) promoted bacterial survival in larvae and may have exerted harmful effects on the hosts. Thus, L-Asp, L-Thr, and L-Tyr putatively controlled bacterial burden in an innate immunity context.

### 3.2. L-Asp, L-Thr, and L-Tyr Do Not Affect Zebrafish Larvae Development

**Figure 2:**
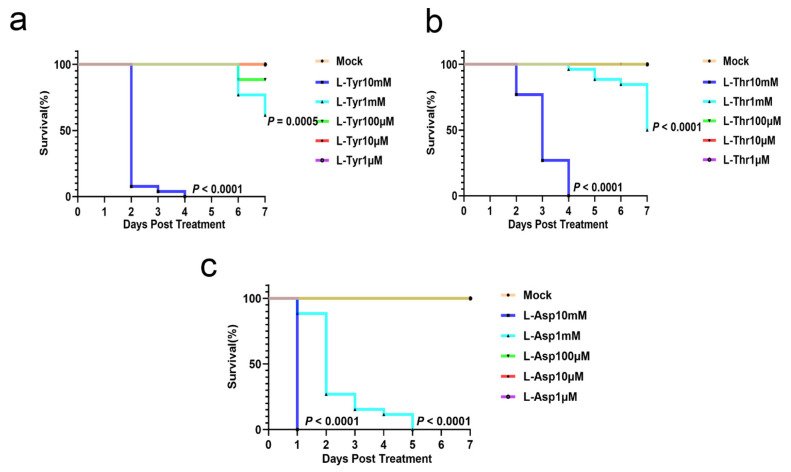
L-Asp, L-Thr, and L-Tyr do not affect zebrafish larvae development. Larvae mortality rates when exposed to different L-Tyr (**a**), L-Thr (**b**), and L-Asp (**c**) concentrations from 0 to 7 days post- fertilization (n = 26/group). The Kaplan–Meier method (**a**–**c**) was used for analysis.

Owing to the long-term toxic side effects of anti-TB drugs, AA supplementation may cause deleterious side effects [31]; therefore, we evaluated AA toxicity in zebrafish larvae. During larval screening, we administered different AA concentrations of L-Asp (1 μM–10 mM), L-Thr (1 μM–10 mM), and L-Tyr (1 μM–10 mM) to the larvae, after which larvae survival was continuously monitored for 7 days. Dose-dependent toxicity effects were observed for L-Asp and L-Thr concentrations > 1 mM and L-Tyr concentrations > 100 μM. These AA treatments were not associated with larva deaths at the concentration of 10 μM, which was estimated for efficacy (Figure 2a–c).

### 3.3. L-Tyr Reduces M. marinum Levels in Adult Zebrafish Granulomas

**Figure 3:**
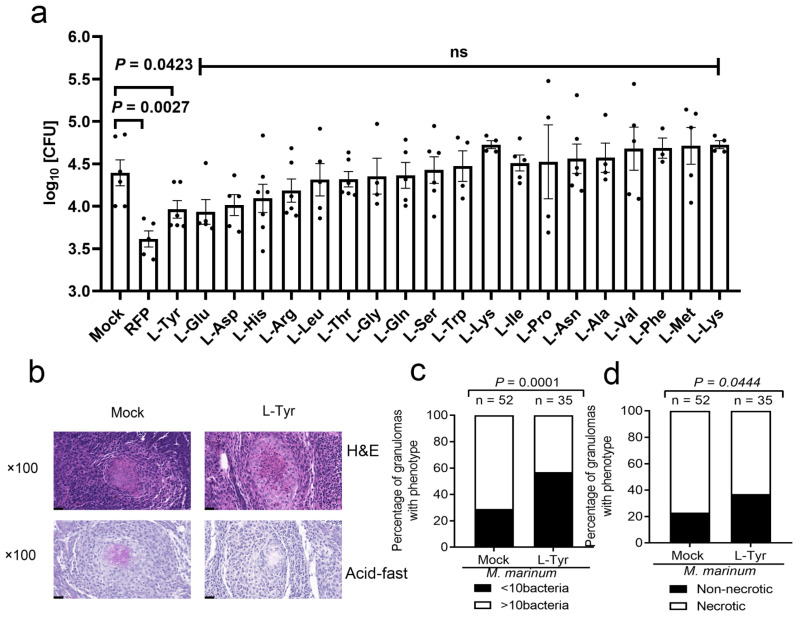
L-Tyr reduces *M. marinum* levels in adult zebrafish granulomas. (**a**) Adults were intraperitoneally infected with approximately 400 CFUs of wild-type *M. marinum* strain/fish for 14 days. Adults were orally administered daily with 20 amino acids (effective concentrations) for 1 week before infection and for 2 weeks after infection. As a positive control, at 7 days post-infection (dpi), fish were orally administered RFP (50 mg/kg) daily until 14 dpi. (**b**) Histopathology showing whole fish sections and bacterial titers. Hematoxylin and eosin or acid-fast staining shows zebrafish infected for 14 days and granuloma comparisons between *M. marinum*-infected adults treated with/without L-Tyr and scored for *M. marinum* burden (> or <10 bacteria) or (**c**) necrotic granuloma percentages in fish (**d**). Individual values are shown with scatter dot plots in Student’s *t*-tests (**a**), and Fisher’s exact tests (**c**,**d**) were used for analyses. Scale bar = 20 μm in (**b**).

When adult zebrafish are infected with *M*. *marinum*, they generate well-defined TB granulomas accompanied by typical central necrosis [32]. In our study, we examined the functional relevance of the 20 AAs during mycobacterial infection and tuberculous granuloma formation in adult zebrafish. From previous studies, we selected effective AA concentrations for adult zebrafish treatments (Table S1), with RFP (50 mg/kg) as the positive control [33]. We observed that only L-Tyr (1 mM) significantly reduced bacterial burden in adults and was not as effective as RFP (Figure 3a). The remaining AAs had no impact on the bacterial burden in adult zebrafish. Furthermore, L-Tyr supplementation markedly decreased high-burden or necrotic granuloma fractions in *M*. *marinum*-infected adult zebrafish (Figure 3b–d and Figure S1). Thus, larvae and adult screening confirmed that L-Tyr supplementation limited mycobacterial survival in zebrafish tuberculous granulomas.

### 3.4. L-Tyr Reduces Mtb Intracellular Survival

**Figure 4:**
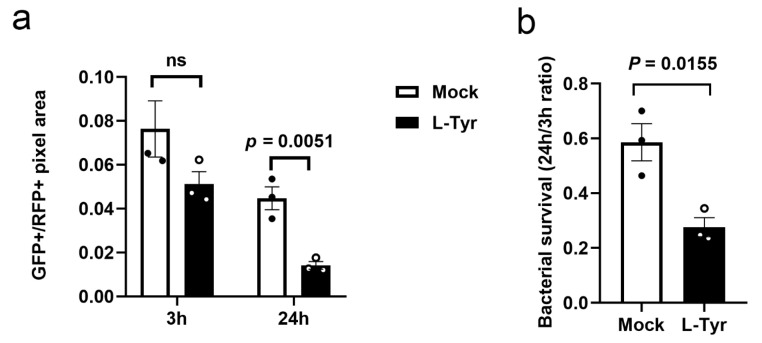
L-Tyr reduces intracellular M. tuberculosis (Mtb) survival. Macrophages pretreated with control or L-Tyr (10 μM) were infected with Mtb at indicated times and subjected to survival analyses (**a**,**b**). Survival rates were estimated by dividing total intracellular GFP-positive signals by total intracellular mCherry-positive signals (**a**). Survival ratios were calculated by dividing the results at 24 h to those at 3 h (**b**). Individual values are shown in scatter dot plots in (**a**,**b**). Student’s *t*-tests (**a**,**b**) were used in analyses.

Macrophage and mycobacteria interplay is the main promoter of granuloma development and tissue dissemination. In our study, we examined if L-Tyr could reduce mycobacteria survival in macrophages. Intracellular Mtb survival in MPMs was compared in resting and L-Tyr-stimulated macrophages. Bacterial viability in live/dead-H37Rv-infected MPMs was enumerated by measuring GFP+/RFP+ fluorescence signals at 3 h and 24 h after infection. After phagocytosis for 3 h, CFUs were equivalent across both conditions (Figure 4a). However, L-Tyr treatment significantly reduced Mtb intracellular survival in macrophages (Figure 4a,b); therefore, L-Tyr promoted macrophage-mediated processes, which facilitated bacterial killing.

### 3.5. L-Tyr Upregulates IFN-γ Transcription in M. marinum-Infected Adult Zebrafish

**Figure 5:**
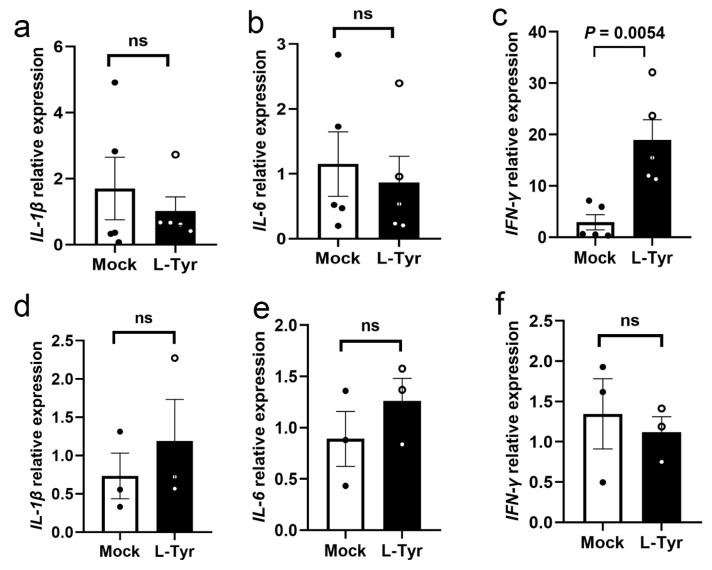
L-Tyr affects proinflammatory cytokine expression. qRT-PCR analysis of IL-1β (**a**), IL-6 (**b**), and IFN-γ (**c**)mRNA expression in controls or L-Tyr (1 mM)-pretreated adult zebrafish intraperitoneally infected with *M*. *marinum*. qRT-PCR analysis of IL-1β (**d**), IL-6 (**e**), and IFN-γ (**f**) mRNA expression in controls or L-Tyr (10 μM)-pretreated zebrafish larvae infected with *M*. *marinum*. Individual values are shown in a scatter dot plot in (**a**–**f**). Student’s *t*-tests (**a**–**f**) were used for analyses.

To examine mechanisms whereby L-Tyr inhibited mycobacterial survival and granuloma progression, we examined proinflammatory cytokine expression. L-Tyr supplementation had no significant effects on interleukin-1β (IL-1β), IL-6, and IFN-γ cytokine expression in MPMs under normal conditions (Figure S2). Next, using qRT-PCR, we examined IL-1β, IL-6, and IFN-γ expression in adult and larvae zebrafish treated with L-Tyr and infected with *M*. *marinum* (Figure 5a–f), with infected and untreated fish as controls. L-Tyr supplementation significantly increased IFN-γ expression in adults, but no other cytokines were affected. However, in larvae, which lacked adaptive immunity, no significant proinflammatory cytokine enhancement was observed. We hypothesized that, as part of adaptive immunity, L-Tyr treatment may have limited mycobacterial survival in adult zebrafish tuberculous granulomas by promoting IFN-γ expression in T lymphocytes.

### 3.6. L-Tyr Inhibits Mtb Intracellular Survival via ROS

**Figure 6:**
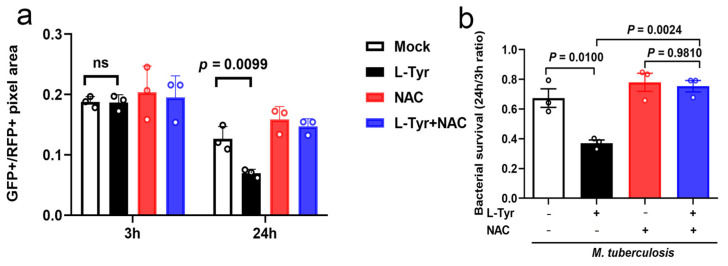
L-Tyr inhibits *M*. *tuberculosis* (Mtb) intracellular survival via ROS. Macrophages pretreated with L-Tyr (10 μM) or NAC (5 mM) were infected with Mtb at indicated times and subjected to survival analyses. MPMs and Mtb residents were imaged using fluorescence microscopy. Survival rates were calculated by dividing total intracellular GFP-positive signals by total intracellular mCherry-positive signals (**a**). Survival ratios were calculated by dividing the results at 24 h to those at 3 h (**b**). Individual values are shown in a scatter dot plot in (**a**,**b**). Student’s *t*-tests (**b**) were used for analyses.

To identify mechanisms whereby L-Tyr inhibited Mtb survival in macrophages, we examined if L-Tyr inhibited intracellular survival associated with the main bactericidal effects of ROS of macrophages using NAC [34]. After NAC treatment, Mtb intracellular survival was promoted, while L-Tyr supplementation had no significant effects on intracellular bacterial burden in NAC-treated macrophages (Figure 6a,b). Therefore, L-Tyr appeared to inhibit mycobacterial survival in macrophages by promoting ROS production.

## 4. Discussion

TB is a significant infectious disease that seriously impacts public health. Its long treatment cycles and high incidence rates require that new therapies be identified. The ability of pathogens to respond to stimuli in the environment is of critical importance for niche adaptation. As an extremely successful intracellular pathogen, mycobacteria must adapt to harsh environments during their evasion of host immune defenses, including hostile granuloma microenvironments. It has been postulated that granuloma microenvironments can modify AA metabolism in immune cells at the expense of bacterial persistence in order to protect against immunopathology [13]. Mtb uses AA uptake and degradation pathways to survive and thrive in hosts. The pathogen extracts essential nutrients from host tissue and cells, thus fulfilling its own metabolic requirements [35]. Studies have reported that the concentrations of common metabolites methionine, asparagine, cysteine, threonine, serine, tryptophan, leucine, citrulline, and phenylalanine are significantly decreased after mycobacterial infection [36,37]. L-Asp is the main nitrogen source used by bacteria when infecting hosts [38]; L-Arg is the biological precursor of NO and is considered a potential anti-TB therapeutic [39]. We showed that, out of 20 proteinogenic AAs, only L-Tyr supplementation simultaneously reduced bacterial burden levels in zebrafish larvae and adults and mycobacteria survival in macrophages. Mechanistically, L-Tyr may limit mycobacterial survival in adult zebrafish tuberculous granulomas by promoting IFN-γ expression in T lymphocytes as part of adaptive immunity responses. In macrophages, L-Tyr may inhibit Mtb intracellular survival by promoting ROS. These results may provide a new potential HDT to combat TB.

*M*. *marinum* is closely related to the Mtb complex and causes a disease called fish TB. In zebrafish larvae, *M*. *marinum*-infected macrophages generate early granulomas, which undergo typical epithelioid transformation and activate granuloma-specific gene expression [40]. *M*. *marinum*-infected adult zebrafish develop well-defined TB granulomas with typical central necrosis [32]. We exploited these characteristics to screen for potentially valuable AAs in treating mycobacterial disease. Surprisingly, L-Arg, which is required for immune cell function [14,15,16,17,18], had no significant effects on bacterial burden and the proportion of high-burden or necrotic granulomas in zebrafish larvae and adults. Moreover, a double-blind trial of oral vitamin D and L-Arg in 200 sputum smear-positive patients reported that vitamin D or L-Arg supplementation had no significant effects on TB outcomes [41]. Our results suggest that L-Arg as a potential therapeutic or preventative measure should be examined in more comprehensive Mtb infection models, especially in animals with typical tuberculous granuloma formation. In our *M*. *marinum*-infected zebrafish larva model, L-Thr and L-Asp significantly inhibited the bacterial burden but had little effect on *M*. *marinum* levels in adults. As we only treated adults with AA concentrations recommended by previous studies, further research is required to clarify if L-Thr and L-Asp have roles in adult zebrafish. L-Ile also significantly enhanced the bacterial burden but had little effect on *M*. *marinum* levels in adults. It was reported that L-Ile effectively induced β-defensins and promoted Mtb elimination in a mouse model, in contrast to our results. Furthermore, L-Ile significantly decreased inflammatory areas in mice infected with drug-sensitive or multidrug-resistant strains [42], which may be unfavorable for controlling the bacterial burden in zebrafish larvae. It was also reported that inhibitory effects toward cancer growth were observed when L-Val was used. Possible mechanisms may have involved altered intracellular protein synthesis due to valine deprivation [43]. Elevated valine concentrations were identified in a group with TB infection [10]. However, few studies have reported on the role of valine in TB. More animal model studies are required to examine if L-Ile or L-Val have roles in mycobacterial infection.

L-Tyr is a non-essential AA synthesized from phenylalanine and is used to generate the catecholamines dopamine and norepinephrine, which are important mood, behavior, and cognition modulators and may be depleted under cognitively challenging or stressful conditions. Several studies have indicated that L-Tyr helps prevent cognitive function decline under stressful and cognitively demanding conditions [44,45,46]. While it may not improve memory under resting conditions, L-Tyr supplementation has been shown to reduce memory under acutely stressful conditions [47]. L-Tyr supplementation can also exert effects on phenylketonuria in humans [48], but its role in bacterial infections remains uncharacterized.

For the first time, we showed that L-Tyr reduced mycobacterial survival in vivo in macrophages and controlled tuberculous granuloma exacerbations. L-Tyr also promoted IFN-γ expression in *M*. *marinum*-infected adult zebrafish but not in larvae. IFN-γ is a key cell-mediated immunity effector and coordinates several anti-microbial functions. As zebrafish larvae lack adaptive immunity, we hypothesized that L-Tyr treatment may have limited mycobacterial survival in adult zebrafish tuberculous granulomas by promoting IFN-γ expression in T lymphocytes as part of an adaptive immunity response to mycobacterial infection. Indeed, IFN-γ is essential for combating mycobacterial infections [49]. CD4 T cells are a major IFN-γ source during adaptive immune responses to Mtb infections [50]. It was previously reported that granuloma metrics in an Mtb-infected Wistar rat model peaked at week 6 and remained consistent between 6 and 12 weeks post-infection. Granuloma kinetics were consistent with IFN-γ levels, which suggests that IFN-γ may be implicated in TB mechanisms [51]. In a *Mycobacterium avium*-infected C57BL/6 mouse model, IFN-γ was required for mycobacteria-induced pulmonary caseous necrosis development [52]. In future work, IFN-γ function in L-Tyr-mediated mycobacterial survival in adult zebrafish tuberculous granulomas must be characterized in an IFN-γ deficient zebrafish model. An important effect of IFN-γ on macrophages is the activation of microbicidal effector functions, especially elevated ROS production and reactive nitrogen species. Accordingly, we also showed that L-Tyr putatively inhibited Mtb survival in macrophages via ROS promotion. When combined, our data suggest that L-Tyr may exert protective roles during mycobacterial infection by strengthening T-cell-mediated, IFN-γ-stimulated ROS production in macrophages. However, the precise mechanisms underlying these regulatory effects require further study.

L-Tyr has considerable potential as an anti-TB AA and may integrate immunometabolism with anti-TB immunity by enhancing T-cell-mediated, IFN-γ-stimulated ROS production in macrophages, thus inhibiting mycobacterial survival in tuberculous granulomas. Unequivocally, novel therapeutic approaches for TB are required, specifically for drug-resistant TB. HDTs are appealing strategies that exploit host–microbe interactions to induce immune responses against Mtb, in contrast to direct bactericidal effects [28]. We provide a novel HDT strategy via the clinical development of AA therapies, which may be effective in active or latent TB patients infected with drug-sensitive or drug-resistant Mtb.

## Figures and Tables

**Table 1:** Study primers.

Primer	Sequence (5′-3′)
β-actin-F	ATGGATGAGGAAATCGCTGCC
β-actin-R	CTCCCTGATGTCTGGGTCGTC
IL-1β-F	TGGACTTCGCAGCACAAAATG
IL-1β-R	GTTCACTTCACGCTCTTGGATG
IL6-F	AGACCGCTGCCTGTCTAAAA
IL6-R	TTTGATGTCGTTCACCAGGA
IFN-γ-F	AAATGGTGCTACTCTGTGGAC
IFN-γ-R	TTCCAACCCAATCCTTTG

## Data Availability

The datasets used and analyzed during the current study are available from the corresponding author on reasonable request.

## Supplementary Materials

The following supporting information can be downloaded at: https://www.mdpi.com/article/10.3390/pathogens12050654/s1. Table S1: Different concentrations of amino acids used in this study. Figure S1: L-Tyr can reduce the amount of *M. marinum* in granuloma of adult zebrafish. Figure S2: L-Tyr had no significant effect on the expression of proinflammatory cytokines under normal conditions. References [31,42,53,54,55,56,57,58,59,60,61,62,63,64,65,66] are cited in the Supplementary Materials.
