# Supplementary material for: L-Tyrosine Limits Mycobacterial Survival in Tuberculous Granuloma
Source: Pathogens. 2023 Apr 28;12(5):654. doi: 10.3390/pathogens12050654 (PMC10221957; doi:10.3390/pathogens12050654)
Supplement: Supplementary file 1 [file pathogens-12-00654-s001.zip › pathogens-2270851-Supplementary Material.pdf]

L-Tyrosine Limits Mycobacterial Survival in Tuberculous Granuloma

Yaxian Gao [1,2,†], Jiaqing Li [1,2,†], Xinya Guo [2], Liru Guan [2], Jie Wang [2], Xiaochen Huang [2], Wenjuan Wang [1,*] and Hua Yang [1,2,*]

[1] The Key Laboratory of Environmental Pollution Monitoring and Disease Control, Ministry of Education, School of Public Health, Guizhou Medical University, Guiyang 550000, China; gaoyaxian@stu.gmc.edu.cn (Y.G.); lijiaqing@stu.gmc.edu.cn (J.L.)

[2] Shanghai Key Laboratory of Tuberculosis, Shanghai Pulmonary Hospital, Tongji University School of Medicine, Shanghai 200433, China; mia.guo@bio-techne.com (X.G.); guanliru_0712@tongji.edu.cn (L.G.); wjtwo@163.com (J.W.); huangxiaochen.1001@163.com (X.H.)

* Correspondence: wangwenjuan@gmc.edu.cn (W.W.); yanghua97065@tongji.edu.cn (H.Y.)

† These authors contributed equally to this work.

The number of supplementary tables: 1

The number of supplementary figures: 2

Supplementary Table

Table S1. Different concentrations of amino acids used in this study

Amino acids	Concentration	References
L-alanine	270 mM	[53]
L-cysteine	3 mM	[54]
L-phenylalanine	150 mM	[55]
L-valine	256 mM	[56]
L-arginine	690 mM	[57]
L-glutamine	68 mM	[57]
L-glutamic acid	68 mM	[58]
L-proline	5 mM	[59]
L-aspartic acid	1 mM	[59]
L-asparagine	1 mM	[60]
L-isoleucine	20 mM	[42]
L-lysine	100 mM	[61]
L-threonine	168 mM	[62]
L-methionine	30 mM	[63]
L-leucine	5 mM	[64]
L-glycine	400 mM	[65]
L-tryptophan	1.5 mM	[66]
L-Tyr (from tyrosine disodi um salt hydrate)	1 mM	[31]
L-histidine	10 mM	
L-serine	10 mM	

Supplementary Figure

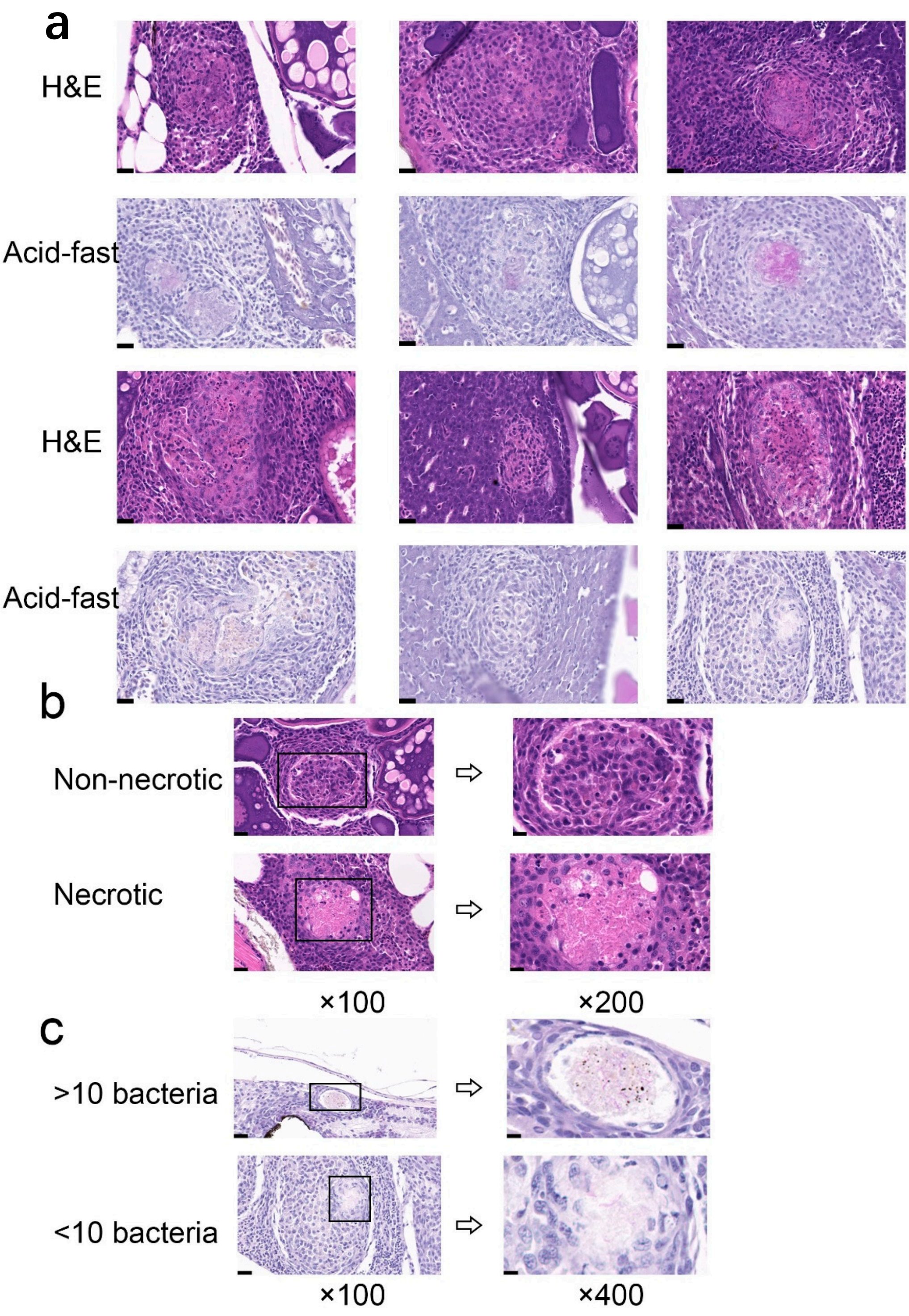

Figure S1. L-Tyr can reduce the amount of *M. marinum* in the tuberculous granulomas of adult zebrafish. (a) The different biological replicate data for H&E and acid-fast staining from adult zebrafish infected for 14 days under Mock or L-Tyr treated condition. (b) The representative pictures of

granulomas with necrotic and non-necrotic granulomas. (c)The representative pictures of granulomas with less than or more than 10 bacteria.

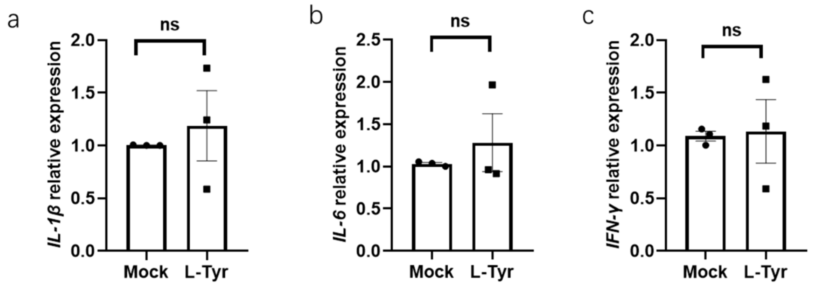

Figure S2. L-Tyr had no significant effect on the expression of proinflammatory cytokines under normal condition. qPCR analysis of IL-1β(a), IL-6(b), IFN-γ(c) mRNA from control or 10 μM L-Tyr pretreated MPM under normal condition. Individual values are shown with scatter dot plot in a- f. Statistical tests: Student's t-test (a-c) was used for statistical analysis.

References

53. Dandare, S.U.; Ezeonwumelu, I.J.; Shinkafi, T.S.; Magaji, U.F.; Adio, A.A.; Ahmad, K. L-alanine supplementation improves blood glucose level and biochemical indices in alloxan-induced diabetic rats. *J. Food Biochem.* **2021**, *45*, e13590. https://doi.org/10.1111/jfbc.13590.
54. Liu, S.; Xin, D.; Wang, L.; Zhang, T.; Bai, X.; Li, T.; Xie, Y.; Xue, H.; Bo, S.; Liu, D.; et al. Therapeutic effects of L-Cysteine in newborn mice subjected to hypoxia-ischemia brain injury via the CBS/H(2)S system: Role of oxidative stress and endoplasmic reticulum stress. *Redox Biol.* **2017**, *13*, 528–540. https://doi.org/10.1016/j.redox.2017.06.007.
55. Jiang, M.; Gong, Q.Y.; Lai, S.S.; Cheng, Z.X.; Chen, Z.G.; Zheng, J.; Peng, B. Phenylalanine enhances innate immune response to clear ceftazidime-resistant Vibrio alginolyticus in Danio rerio. *Fish Shellfish Immunol.* **2019**, *84*, 912–919. https://doi.org/10.1016/j.fsi.2018.10.071.
56. Ma, Q.; Hu, L.; Zhu, J.; Chen, J.; Wang, Z.; Yue, Z.; Qiu, M.; Shan, A. Valine Supplementation Does Not Reduce Lipid Accumulation and Improve Insulin Sensitivity in Mice Fed High-Fat Diet. *ACS Omega* **2020**, *5*, 30937–30945. https://doi.org/10.1021/acsomega.0c03707.
57. Cao, Y.; Feng, Y.; Zhang, Y.; Zhu, X.; Jin, F. L-Arginine supplementation inhibits the growth of breast cancer by enhancing innate and adaptive immune responses mediated by suppression of MDSCs in vivo. *BMC Cancer* **2016**, *16*, 343. https://doi.org/10.1186/s12885-016-2376-0.
58. Rowe, A.A.; Patel, P.D.; Gordillo, R.; Wert, K.J. Replenishment of TCA cycle intermediates provides photoreceptor resilience against neurodegeneration during progression of retinitis pigmentosa. *JCI Insight* **2021**, *6*, 17. https://doi.org/10.1172/jci.insight.150898.
59. Mantilla, B.S.; Marchese, L.; Casas-Sanchez, A.; Dyer, N.A.; Ejeh, N.; Biran, M.; Bringaud, F.; Lehane, M.J.; Acosta-Serrano, A.; Silber, A.M. Proline Metabolism is Essential for Trypanosoma brucei brucei Survival in the Tsetse Vector. *PLoS Pathog.* **2017**, *13*, e1006158. https://doi.org/10.1371/journal.ppat.1006158.
60. Deng, L.; Yao, P.; Li, L.; Ji, F.; Zhao, S.; Xu, C.; Lan, X.; Jiang, P. p53-mediated control of aspartate-asparagine homeostasis dictates LKB1 activity and modulates cell survival. *Nat. Commun.* **2020**, *11*, 1755. https://doi.org/10.1038/s41467-020-15573-6.
61. Schobel, F.; Jacobsen, I.D.; Brock, M. Evaluation of lysine biosynthesis as an antifungal drug target: Biochemical characterization of Aspergillus fumigatus homocitrate synthase and virulence studies. *Eukaryot. Cell* **2010**, *9*, 878–893. https://doi.org/10.1128/EC.00020-10.
62. Gaifem, J.; Goncalves, L.G.; Dinis-Oliveira, R.J.; Cunha, C.; Carvalho, A.; Torrado, E.; Rodrigues, F.; Saraiva, M.; Castro, A.G.; Silvestre, R. L-Threonine Supplementation During Colitis Onset Delays Disease Recovery. *Front. Physiol.* **2018**, *9*, 1247. https://doi.org/10.3389/fphys.2018.01247.
63. Dever, J.T.; Elfarra, A.A. L-methionine toxicity in freshly isolated mouse hepatocytes is gender-dependent and mediated in part by transamination. *J. Pharmacol. Exp. Ther.* **2008**, *326*, 809–817. https://doi.org/10.1124/jpet.108.141044.
64. Wessler, L.B.; Farias, H.R.; Ronsani, J.F.; Candiotto, G.; Dos, S.P.; de Oliveira, J.; Rico, E.P.; Streck, E.L. Acute exposure to leucine modifies behavioral parameters and cholinergic activity in zebrafish. *Int. J. Dev. Neurosci.* **2019**, *78*, 222–226. https://doi.org/10.1016/j.ijdevneu.2019.10.001.

65. Tsuji-Tamura, K.; Sato, M.; Fujita, M.; Tamura, M. Glycine exerts dose-dependent biphasic effects on vascular development of zebrafish embryos. *Biochem. Biophys. Res. Commun.* **2020**, *527*, 539–544. https://doi.org/10.1016/j.bbrc.2020.04.098.
66. Giacomini, A.; Piassetta, A.S.; Genario, R.; Bonan, C.D.; Piato, A.; Barcellos, L.; de Abreu, M.S. Tryptophan alleviates neuroendocrine and behavioral responses to stress in zebrafish. *Behav. Brain Res.* **2020**, *378*, 112264. https://doi.org/10.1016/j.bbr.2019.112264.
